# Approaches Based on Artificial Intelligence and the Internet of Intelligent Things to Prevent the Spread of COVID-19: Scoping Review

**DOI:** 10.2196/19104

**Published:** 2020-08-10

**Authors:** Aya Sedky Adly, Afnan Sedky Adly, Mahmoud Sedky Adly

**Affiliations:** 1 Faculty of Computers and Artificial Intelligence Helwan University Cairo Egypt; 2 Faculty of Physical Therapy Cardiovascular-Respiratory Disorders and Geriatrics, Laser Applications in Physical Medicine Cairo University Cairo Egypt; 3 Faculty of Physical Therapy Internal Medicine Beni-Suef University Beni-Suef Egypt; 4 Faculty of Oral and Dental Medicine Cairo University Cairo Egypt; 5 Royal College of Surgeons of Edinburgh Scotland United Kingdom

**Keywords:** SARS-CoV-2, COVID-19, novel coronavirus, artificial intelligence, internet of things, telemedicine, machine learning, modeling, simulation, robotics

## Abstract

**Background:**

Artificial intelligence (AI) and the Internet of Intelligent Things (IIoT) are promising technologies to prevent the concerningly rapid spread of coronavirus disease (COVID-19) and to maximize safety during the pandemic. With the exponential increase in the number of COVID-19 patients, it is highly possible that physicians and health care workers will not be able to treat all cases. Thus, computer scientists can contribute to the fight against COVID-19 by introducing more intelligent solutions to achieve rapid control of severe acute respiratory syndrome coronavirus 2 (SARS-CoV-2), the virus that causes the disease.

**Objective:**

The objectives of this review were to analyze the current literature, discuss the applicability of reported ideas for using AI to prevent and control COVID-19, and build a comprehensive view of how current systems may be useful in particular areas. This may be of great help to many health care administrators, computer scientists, and policy makers worldwide.

**Methods:**

We conducted an electronic search of articles in the MEDLINE, Google Scholar, Embase, and Web of Knowledge databases to formulate a comprehensive review that summarizes different categories of the most recently reported AI-based approaches to prevent and control the spread of COVID-19.

**Results:**

Our search identified the 10 most recent AI approaches that were suggested to provide the best solutions for maximizing safety and preventing the spread of COVID-19. These approaches included detection of suspected cases, large-scale screening, monitoring, interactions with experimental therapies, pneumonia screening, use of the IIoT for data and information gathering and integration, resource allocation, predictions, modeling and simulation, and robotics for medical quarantine.

**Conclusions:**

We found few or almost no studies regarding the use of AI to examine COVID-19 interactions with experimental therapies, the use of AI for resource allocation to COVID-19 patients, or the use of AI and the IIoT for COVID-19 data and information gathering/integration. Moreover, the adoption of other approaches, including use of AI for COVID-19 prediction, use of AI for COVID-19 modeling and simulation, and use of AI robotics for medical quarantine, should be further emphasized by researchers because these important approaches lack sufficient numbers of studies. Therefore, we recommend that computer scientists focus on these approaches, which are still not being adequately addressed.

## Introduction

With the emergence of the novel coronavirus disease (COVID-19) pandemic, many regions and countries have been facing different risks at different times. Due to the high infectivity and spread of severe acute respiratory syndrome coronavirus 2 (SARS-CoV-2), the virus that causes COVID-19, combined with its increased hospitalization rate, COVID-19 is a serious public health threat worldwide [1,2]. Artificial intelligence (AI) systems such as cognitive computing, deep learning, convolutional neural networks, and machine learning can play a critical role in detection, large-scale screening, monitoring, reduction of caregiver workload, and prediction of possible interactions with the new therapies for this virus [3-6]. In this review, we provide an analysis of AI applications that may help limit the spread of or even help eradicate this virus if properly adapted and applied by countries.

## Methods

We conducted an electronic article search using the MEDLINE, Google Scholar, Embase, and Web of Knowledge databases. The search included only English-language publications, with a focus on evidence-based research articles. The search focused on both randomized and nonrandomized controlled trials, longitudinal studies, cross-sectional studies, and retrospective studies. A list of keywords were used with different combinations, as follows: *SARS-CoV-2*, *COVID-19*, *novel coronavirus*, *artificial intelligence*, *internet of things*, *telemedicine*, *machine learning*, *modeling*, *simulation*, and *robotics*.

## Results

**Figure 1:**
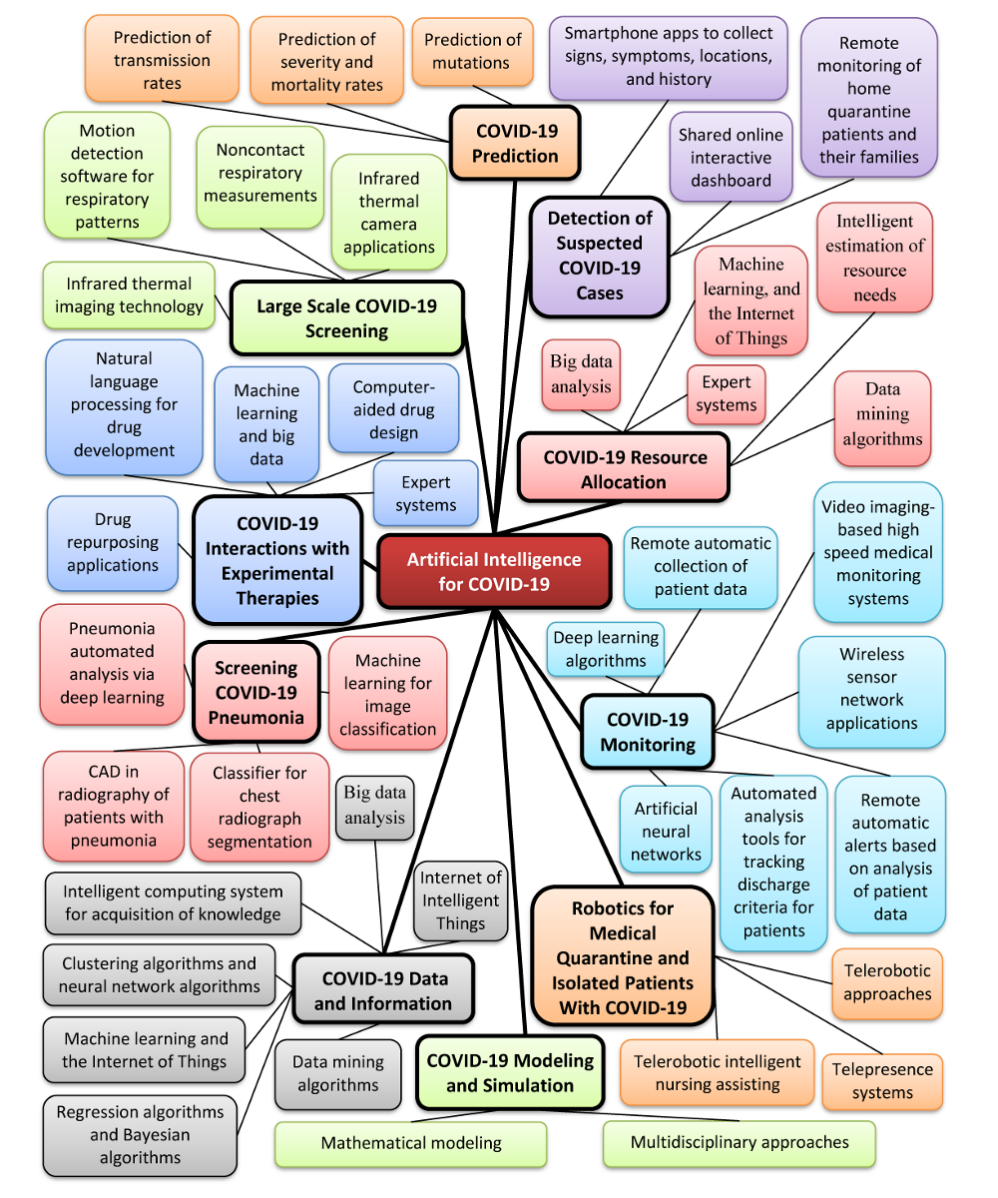
Summary of AI approaches to address the COVID-19 pandemic. AI: artificial intelligence; CAD: computer-aided diagnosis; COVID-19: novel coronavirus disease.

Figure 1 shows a summary of AI-based approaches to prevent the spread of COVID-19.

### AI for Detection of Suspected COVID-19 Cases

**Figure 2:**
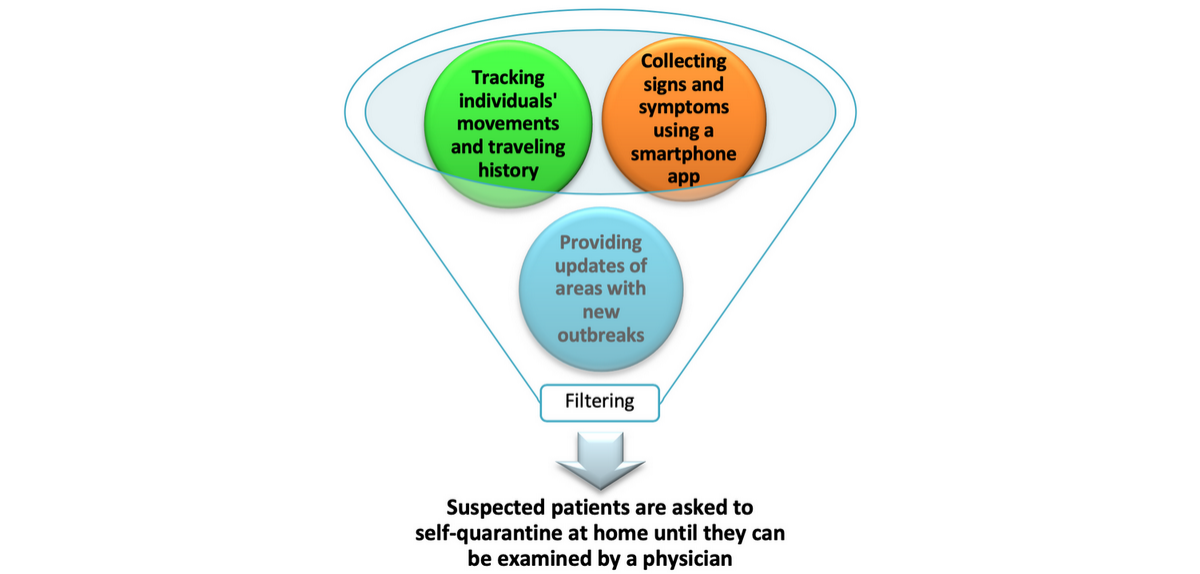
Potential artificial intelligence–based detection of suspected patients with coronavirus disease using a smartphone app.

A pandemic creates unique challenges to the delivery of health care, and these challenges must be faced by a limited number of health care workers. AI can help address these problems; for example, a smartphone app could be developed that collects signs, symptoms, previous locations of the patient, travel history, and updated areas of the outbreak, then processes and filters this information using algorithms so that only suspected cases are examined by physicians (Figure 2) [7].  

On January 22, 2020, the Center for Systems Science and Engineering at Johns Hopkins University launched a publicly shared web-based interactive dashboard [8]. The aim of this dashboard was to accurately visualize and track reported cases of COVID-19 in real time. This revolutionary idea has contributed to the rapid identification of new outbreaks of the disease. The information on this dashboard is updated twice daily, which provides an added advantage. This has opened a new spectrum of AI to predict and recommend quarantine in areas where a threshold number of cases is reached. It can also aid early diagnosis of patients if they report travelling to these areas. All these data are displayed freely through Google Sheets and the ArcGIS Living Atlas [9].

Another important application of AI is remote monitoring of home-quarantined patients and their families via smartphones or smart bracelets. An automatic alarm will sound and provide a warning message if the patient or family member breaks quarantine.

All these contributions can significantly decrease the burden on health care workers and enable them to work more efficiently in a safe environment [7].

### AI for Large-Scale COVID-19 Screening

Noncontact systems that use AI to measure signs and symptoms of COVID-19 are extremely important for large-scale screening in a significantly short period (Table 1). Rapid screening using these systems not only enables remote detection of suspected cases but can also be performed by significantly fewer personnel, which can reduce the work stain on areas such as airports and supermarkets and further augment social distancing [7].

Noncontact systems that can be used for large-scale screening include infrared thermal imaging technology and camera-based motion detection software to detect and analyze abnormal respiratory patterns [10].

One of the diagnostic signs of COVID-19 is fever [10]. Infrared thermal cameras enable real-time visualization of any transient or constant changes in the energy radiating from individuals, which enables estimation of surface temperature [11]. With the aid of AI detection algorithms, suspected individuals with COVID-19 can be automatically identified and tracked using infrared thermal cameras with minimal need for human monitoring.

Respiratory pattern is another diagnostic sign of individuals with COVID-19 [12,13]. The pattern of respiration in COVID-19 is quite different from that in the common cold or influenza [14,15]. Tachypnea, or abnormally rapid breathing, may indicate infection with COVID-19. Using a depth camera to conduct noncontact respiratory measurements of individuals and analyzing these measurements using AI detection algorithms was found to be a promising method for additional confirmation of COVID-19 cases [12,13].

**Table 1:** Comparison of the cost and efficiency of AI systems versus conventional human labor for large-scale screening of COVID-19.

Parameter	AI^a^ systems for COVID-19^b^ screening	Conventional human labor for COVID-19 screening
Cost	May be initially high but significantly decrease with time, leading to lower cumulative cost.	May be high or low according to country but will generally have higher cumulative cost, including the added cost of applying preventive measures for direct contact.
Sensitivity (probability of detection)	Very high if multiple confirmatory methods are used.	Affected by distractors.
Specificity (excluding negative conditions)	False positive results may occur due to other conditions having similar signs.	When screening is performed by expert health care workers, specificity will be high.
Duration of screening	Very short.	Relatively long and may require additional employees to shorten the duration.
Number of working hours	24 hours, 7 days a week in addition to the working hours of quarantined physicians and health care workers who are suspected to have COVID-19, who will be able to work from home.	No more than 48 hours of work per week and no more than an average of 8 hours of nighttime work per 24 hours of total work [16].
Possibility of COVID-19 infection among examined subjects and examiners	Very little or no contact between persons, which significantly decreases the possibility of transmission of COVID-19 infection.	High risk of transmission of COVID-19 infection even if preventive measures are followed, as human error may occur.

^a^AI: artificial intelligence.

^b^COVID-19: coronavirus disease.

### AI for COVID-19 Monitoring

Large amounts of data from patients with COVID-19 are routinely manually collected and interpreted in hospitals with or without standalone monitoring devices. The processes of collecting patients’ monitor data in hospital wards are different in many hospitals, as different approaches are used worldwide. In some hospitals, data are only collected manually and then recorded in spreadsheets, which are discarded after the patient is discharged [17].

AI systems can collect monitor data from patients using personal digital assistants (PDAs), tablets, and similar equipment; the patients’ data can be stored in electronic health records (EHRs), where they can be easily shared and rapidly transferred when needed [18].

With different technologies and AI, the collection, analysis, and interpretation of patients’ monitor data can be fully or partially automatized, which diminishes both infection risk and the burdens imposed on medical staff to constantly gather, store, analyze, and interpret these data [17].

Several intelligent and expert systems have been developed for medical monitoring using wireless sensor networks. These methods have been found to be efficient when signals are continuous; however, the applicability of these expert systems to incomplete data has not yet been established [19].

Thoracic computed tomography (CT) without contrast is considered to be an effective method to detect, quantify, and monitor symptoms of patients with COVID-19. Deep learning algorithms could be developed to contribute to the analysis, interpretation, and tracking of large numbers of thoracic CT examinations [20].

Developing a video imaging–based high-speed medical monitoring system that uses motion tracking monitoring algorithms for patients with COVID-19 can also provide a large amount of factual information regarding vital signs (temperature, heart rate, respiratory rate, blood pressure, oxygen saturation) as well as status, condition severity, existing comorbidities, and patient discharge. Modal parameters can be subsequently extracted to analyze the level of severity or damage from COVID-19. Motion tracking monitoring has been investigated in several studies with very promising results [21,22].

It has been reported that patients with COVID-19 can be discharged from the quarantine ward if they meet the following criteria: being afebrile for a minimum duration of three days, respiratory symptoms resolved, improvements in radiological signs for pulmonary infiltrates, at least two consecutive negative COVID-19 nucleic acid tests with sampling intervals of at least 24 hours [15].

AI-based automated analysis tools for tracking the discharge criteria for patients with COVID-19 should be developed to determine and differentiate treated patients from those who still need to be isolated. In addition, AI-based automated CT image analysis tools for tracking, quantification, and detection of COVID-19 can assist in differentiating patients with COVID-19 from individuals who do not have the disease. Moreover, artificial neural networks can be trained to infer qualitative characteristics that are based on intensity as well as network inferences, which can be correlated according to the patient’s condition.

An exploratory study showed that AI can reliably and efficiently contribute to accurate detection and tracking of the progression or resolution of COVID-19. In this work, it was also reported that the rapid developments in AI-based automated tools for CT image analysis can achieve a high degree of accuracy in detection of COVID-19–positive patients in addition to precise assessment of the severity of the disease [23,24].

### AI for Analysis of Experimental Therapies for COVID-19

Computer-aided drug design is highly efficient for rapidly identifying drug repurposing candidates and should be further investigated.

One example is the mechanism-based inhibitor N3, which was identified using computer-aided drug design. N3 was found to particularly inhibit the main protease of SARS-CoV-2 because it can fit within a substrate-binding pocket [25].

A crucial application for computer-aided drug repurposing is treating novel diseases such as COVID-19 by identifying drugs that were developed to treat other diseases. Drug repurposing can be realized by conducting systematic drug-drug interaction analyses and studying drug-target interactions, which can be performed using AI-based algorithms [26-28].

AI has been recognized to have a transformative influence on drug development. In accordance with a recent report, machine learning and big data can have great effects on health care systems and may have positive outcomes in the pharmaceutical market, as it has been predicted by industry experts that developing drugs through AI methods will provide significantly improved feedback. However, AI-based drug development may be slower to launch in the long term. If applied correctly, these methods can be highly competitive in the pharmaceutical industry [29,30].

Natural language processing is another branch of AI that can be applied in COVID-19 drug development. Its methods can be beneficial for extracting meaning from text using machine learning approaches and searching for external biomedical content in drug discovery [31,32]. Another AI approach is machine or computer vision, which is the use of algorithms to enable computers to comprehend the content of images. These images can be used to understand cell anatomy and identify novel treatments for COVID-19 [33].

The use of AI is prevalent in drug discovery, and many pharmaceutical corporations have established in-house partnerships or initiatives with AI companies. Some organizations are currently using AI approaches to find novel uses for late-stage drug candidates or repurpose existing drugs [34,35]. However, data of sufficient quality are needed to train systems for COVID-19 AI implementation. Data accessibility is an additional challenge because the systems will be trained via supervised learning, which requires substantial amounts of data on COVID-19 to accurately perform complex tasks.

Accessibility of data can also create costs if these data must be accessed by corporate partners or technology providers. Additionally, the data should be of high quality, as poor or corrupt data can affect the results. At present, although industry standards for data have been established for many uses, they currently may not apply in the case of COVID-19. Moreover, a substantial amount of effort is required to integrate data on COVID-19 into corporation systems to use it for AI.

### AI for Screening COVID-19 Pneumonia

COVID-19 is spreading very rapidly worldwide; meanwhile, it can be difficult to diagnose the disease. These limitations can be partially accredited to the limited number of physicians who are capable of interpreting data and using identification methods compared to the great increase in the number of cases [36].

It has been reported that radiologists can distinguish COVID-19 pneumonia from other types of pneumonia in chest CT images with high specificity. Other reports have demonstrated that reverse transcriptase–polymerase chain reaction (RT-PCR) analysis has a low sensitivity of about 60% to 71% for detection of COVID-19 [37]. This may be due to decreased viral load in the test specimen or to laboratory error. The false negatives produced by RT-PCR analysis can hinder quarantine efforts and require tests to be repeated. On the other hand, the sensitivity of chest CT has been established to be approximately 56% to 98% for COVID-19 detection at initial presentation, and it may help rectify false negative results that are obtained by RT-PCR analysis during the early stages of development of the disease. Additionally, chest CT images can reveal disease progression [33].

Compared to non–COVID-19 pneumonia, CT images of COVID-19 pneumonia are more likely to show vascular thickening, fine reticular opacity, ground-glass opacity, peripheral distribution, and reverse halo sign in addition to bilateral peripheral involvement of multiple lobes. The CT signs may improve gradually approximately 14 days after the onset of symptoms. On the other hand, CT images of COVID-19 pneumonia are unlikely to show both peripheral and central distribution, pleural effusion, pleural thickening, lymphadenopathy, or air bronchogram [38]. Thus, future directions in chest CT may involve developing an AI-based classifier that can further augment the performance of radiologists when combined with clinical information.

Exploration of automated pneumonia analysis via deep learning is a very important issue for examination for many different reasons. The most important reason is that the chest radiographs of COVID-19 patients must be reviewed by highly trained specialists, which creates large amounts of work for those specialists. Further, it is very difficult to read these images because pneumonia is normally revealed over one or more areas of increased opacity [39]. This increase may be due to a reduction of the ratio of gas to soft tissue (lung parenchyma, stroma, and blood) in the lungs. Thus, when an amplified attenuation area (opacification) is reviewed on a CT or chest radiograph, it is crucial to define where the opacifications occur. Diagnosis is very complicated because other conditions can alter the appearance of a radiograph, such as bleeding, surgical changes, post-radiation changes, pulmonary edema, lung cancer, and volume loss due to collapse or atelectasis; aspects such as inspiration depth and patient positioning may also affect the radiograph. Due to these issues, interpreting chest images of COVID-19 patients by the human eye alone is extremely challenging [38].

Although the fields of machine learning and pneumonia research are individually well developed, very limited work is currently available regarding the application of machine learning for diagnosis of pneumonia, especially for patients with COVID-19. Research on deep learning algorithms has recently shown rapid progress. In general, pneumonia diagnosis for medical fields is a broadly known discipline. The combination of those two areas would be novel and promising [40]. Previous research has been performed to apply image processing techniques for identification of cases with pneumonia. Other algorithms have been developed to crop and extract the lung regions from images. The Otsu thresholding method has been used to segregate infected cloudy pneumonia lung regions from healthy regions. Another detection method has been proposed based on cellular neural networks; the simulated results showed remarkable performance in differentiating the lung region area and normal area based on changes in segmentation and grayscale color [41].

It was also reported that machine learning has the important advantage of increased capability to evaluate the effects of interventions when studying subpopulations in clinical pneumonia research [40]. Another significant aspect of machine learning for identifying COVID-19 infection is image classification. This has resulted in the development of a range of networks for image classification. These networks usually employ backpropagation algorithms. Very large neural networks are usually needed for deep learning architectures. Some commonly used deep learning architectures include GoogLeNet, ResNet, and AlexNet [42].

Computational models for deep learning can discover intricate structures in very large data sets by using backpropagation algorithms to indicate how a machine would modify the internal parameters it uses to compute representations of each layer from representations of the previous layer [43]. Training deep learning networks that can recognize symptoms of COVID-19 pneumonia would be very beneficial. These networks could facilitate prescreening and automated diagnosis.

Research has provided insights into testing difficulties associated with very large radiograph image data sets. Most research in this area focuses on small and controlled data sets. For these data sets, irregular features may not be an issue. However, as the size of the data set increases, the number of irregular images also increases. It is particularly important to overcome this potential hurdle for COVID-19 diagnosis because a model that is used by medical industries must be capable of considering all radiographic forms. However, the model will still be required to function at high levels regardless of whether these image types are imputed [42].

Applying computer-aided detection to radiographs of patients with pneumonia may provide a supplement or alternative to human reading [44]. To develop an efficient AI algorithm that uses chest radiographs to predict clinical outcomes, the clinical settings and disease burden profiles of COVID-19 prevalence should be considered.

Software should be trained to use selected chest radiographs of patients with COVID-19 to automatically segment fields of the lung in other radiographs. After the classifier is trained on randomly selected chest radiographs, it can be applied to the remaining chest radiographs.

Chest radiographs commonly demonstrate many categories of abnormalities. Some disease processes may show a combination of abnormal shapes where the disease process has altered the patient’s normal anatomy, such as cardiomegaly and focal abnormalities. These focal abnormalities may appear as isolated density changes, such as pulmonary nodules and texture abnormalities. Texture abnormalities are characterized by diffuse changes [45]. In these situations, texture analysis may be useful for assigning a probability to each location in the lung fields [39].

In general, further developments and validation should be performed in multicenter studies, which would be significant for future research on AI and computer-aided diagnosis in COVID-19 radiology.

### AI and the Internet of Intelligent Things for COVID-19 Data and Information Gathering and Integration

Patients with COVID-19 can provide novel types of data that are relevant to achieving medical goals. Self-tracking devices, mobile health apps, and social media can provide patients with information about COVID-19 and can enable them to monitor their health or even achieve a certain goal. Incorporating AI into devices offers tools and methods for designing and analyzing therapies that can be made accessible to patients and clinicians while supporting consistent integration into patients’ lives and clinicians’ practices [17,46,47]. This field not only offers promising challenges for patients with COVID-19 but can also improve systematic data collection to determine treatment effectiveness.

In the context of the Internet of Intelligent Things (IoIT), a “thing” is a system or entity composed of subsystems, and every subsystem is an indispensable component of the system [48]. Thus, if we divide COVID-19 medical and diagnostic devices in health centers or hospitals into things and modulate these things to be sufficiently intelligent to operate on their own, we can establish a behavior for every subsystem. The challenge is to understand each thing separately. Therefore, to understand the behavior of things used to help control COVID-19, it is necessary to understand the main subcomponents of each thing; then, we must understand the behavior of each subsystem to understand the behavior of the thing as a whole and how to enable it to connect and communicate with other things via the internet.

A crucial challenge for the advancement of digital health is efficient and effective integration of incomplete or heterogeneous information about COVID-19 from different sources and different types, such as interoperability solutions, insufficient availability, and existence of current information silos. All this contradicting information is hindering the development of effective applications. Different information types must be integrated, such as clinical information (including EHRs), information extracted from the biomedical literature by text mining, and high-throughput information on how drugs or chemicals interact in different circumstances [36].

Additionally, big data may be useful to optimize resource use when making informed decisions based on the availability of data related to COVID-19 cases. AI, machine learning, and the internet of things (IoT) could contribute significantly to this process [49-51].

Big data analysis performed via AI can be interpreted as a means of training computers to mimic thinking patterns and even simulate human behaviors. It is assumed that the accuracy of the achieved results will increase with increasing availability of processing power and data because it is desirable to perform machine learning using real-time data [52,53].

Intelligent computing systems can support decision-making even when the problems are complex. A great deal of success has been achieved in integrating expert systems into intelligent systems. However, expert systems may face difficulties in acquiring and processing COVID-19 knowledge. Thus, to recognize the involved patterns and the knowledge gained from different fields, it is vital to combine data mining with intelligent computing systems to determine the information gathered and the patterns involved, which may include clustering algorithms, neural network algorithms, regression algorithms, and Bayesian algorithms [54].

Numerous other challenges may also emerge, including privacy breaches, ethical concerns, and lack of information security. Thus, the ability to share, analyze, and gather information about COVID-19 in real time with different devices may add to the difficulty of maintaining patient privacy.

The mining of very large COVID-19 data sets may present difficulties in terms of computation and storage. For example, combining various types of information in heterogeneous COVID-19 data sets with global information systems can be complicated. Additionally, numerous COVID-19 experts are needed to formulate the data mining process. Finally, the accuracy of data mining results depends on the level of diversity of the gathered COVID-19 data set.

Data mining can also provide many benefits. For example, powerful high speed processes can be established to examine the enormous amounts of information related to COVID-19 and can provide a knowledge base for a specific area of COVID-19 information. Additionally, the diagnosis and prediction of COVID-19 can be automated, and data mining can enhance decision-making processes.

### AI for Resource Allocation to Patients With COVID-19

As more countries are affected by COVID-19 worldwide, it is increasingly necessary to prioritize allocation of resources to patients with the disease [55].

If we attempt to calculate conservative estimates of the resources that will be needed to fight COVID-19, we find that the resources needed are beyond the available capacities of health care facilities. To achieve an accurate estimation of these resources, estimations should include human resources (such as medical staff, including therapists and nurses) and other facilities (including numbers of hospitals, emergency departments, intensive care units, adult beds, neonatal beds, pediatric beds, ventilators, oxygen concentrators, oxygen cylinders, oxygen plants, liquid oxygen, medications, personal protective equipment, critical medical supplies, and pulse oximeters). It is very important to assess all these resources before establishing any action plan for resource allocation.

Given these numbers, if the curve of infected individuals is not reduced and flattened over a short period of time, the COVID-19 pandemic will likely result in a shortage of medical resources, particularly ventilators and hospital beds. The number of medical workers who will be affected should also be taken into consideration, as these workers will commonly be quarantined.

In addition, even after a vaccine is developed, time will be needed to produce, distribute, and administer it; shortages of the vaccine will probably arise as well.

Supply limitations restrain the speed of producing more resources, such as ventilators. Currently, manufacturers cannot state with certainty how many ventilators they can produce during a year.

Policy makers are facing decisions about which patients will be provided with available resources while taking into consideration that patients who do not receive these resources will very likely die.

These decisions cannot be made based on age alone. Professionals have indicated that prioritization should be based on which patients are more likely to survive to save the maximum possible number of lives [56].

To address the COVID-19 resource allocation problem, it is necessary to understand complex data structures related to the prioritization criteria and resources in question.

Applying big data analysis and data mining algorithms, including unsupervised and supervised machine learning, to optimize the prioritization process would be very helpful to reduce harm as much as possible in emergency situations when resources are scarce. With the use of a trained model, machine learning can also minimize human effort while improving or maintaining accurate prioritization.

Moreover, intelligent estimation of resource needs (especially oxygen needs) is crucial to be as accurate as possible in addition to deciding which source of oxygen would be better for a patient by considering the total gross oxygen flow that would be needed through anticipating the load of each patient based on severity and number of patients.

Developing an expert system or framework for reasonable and fair allocation of COVID-19 therapeutics and resources is very complex and requires coordination of many variables and estimations that must be guided by comprehensive and accurate information based on IoT technology, which can provide information about distributions, numbers, sizes, capacities, and risks related to both resources and the affected populations worldwide. The realization of such frameworks or expert systems will mainly depend on sharing and collecting data; this can be greatly facilitated by the IoT.

### AI for COVID-19 Prediction

#### Transmission Rates

In the initial stages of the COVID-19 outbreak, it is critical to analyze and understand the dynamics of transmission of the virus. Changes in the estimations of transmission over time can offer insights in epidemiological situations and demonstrate the effectiveness of outbreak control measures [57].

These analyses can also provide predictions of future growth, assist risk estimation for countries, and guide strategy for alternative interventions [58]. These analyses present many challenges, especially in real time. Moreover, with COVID-19, the appearance of symptoms may be delayed due to the period of incubation; also, confirmation of cases may be delayed in accordance with detection and testing capacity.

AI approaches can account for these delays as well as for uncertainty by explicitly incorporating delays that result from the natural history of virus infection or reporting processes. In addition, individual data sources may be incomplete, be biased, or only capture some aspects of the dynamics of the outbreak. However, evidence synthesis approaches that fit with multiple data sources can enable more robust estimations of the transmission dynamics that can be gathered from noisy data [58,59].

Many factors can affect the transmission dynamics of COVID-19 throughout a country, such as outflow population size from a certain place to affected provinces or cities, geographic locations, interventions, social and economic activities, health care facilities, and environmental heterogeneity. The process of clustering temporal dynamics will provide various insights into the patterns of COVID-19 propagation. In addition, modified auto-encoders have been used to predict the accumulative number of new confirmed cases of COVID-19. By hypothesizing the initial amounts of the epidemical outbreak, modified autoencoders can be used with known architectures and parameters to predict the sizes of future outbreaks and simulate the impact of interventions on the severity and size of epidemics [43,57,60].

Data-driven AI-based methods offer real-time forecasting techniques for estimating and tracking the severity of epidemics, assessing their trajectory, predicting their length, and supporting decision-making by health care workers and governments [61,62].

#### Mutations

The prediction of genetic mutations in the SARS-CoV-2 genome has attracted much attention. Rapid progress has been realized to predict these mutations and analyze their effects. Tracing the mutations of SARS-CoV-2 can provide comprehensive understanding of the evolution dynamics of the virus.

In some studies, antigenic cartographies have been developed for quantifying and visualizing site mutations and antigenic differences [63]. Neural networks were applied in another study to predict point mutations that may appear on structure alignments of primary RNA sequences [64]. Network models were also outlined to demonstrate the dynamics and evolutionary patterns of a virus [65].

Many RNN-based neural networks have been developed for predicting time series tasks [66]. K-means clustering can also be used to find clusters of mutations of SARS-CoV-2, which can provide insights into the nature of mutations and how they can be addressed.

A model was proposed to forecast the properties of viruses that are not characterized antigenically using phylogenetic trees. Modeling sequential data dynamically is important. Recent research has provided ways to embed biological sequences into lower-dimensional vector spaces [67].

#### Severity and Mortality Rates

The assessment of COVID-19 severity by clinical presentation can no longer meet urgent clinical needs. Thus, introducing a deep learning–based model by quantitating clinical features to predict the severity and mortality rates of COVID-19 will be of significant value. Deep learning–based quantitative CT measurements of the extent of lesions and clinical features on initial admission can assist in predicting COVID-19 severity; this will enable physicians to triage patients and design treatment protocols and follow-up evaluations in advance.

Convolutional neural networks were introduced as a potential solution to problems faced in automatic organ segmentation [57,68].

In a recent study, a new model to forecast the prognosis of COVID-19 was established. It has been reported that parsimonious models, which contain five features (age, lactate dehydrogenase, C-reactive protein, CD4^+^ T-cell counts, and mass of infection), are an ideal measure for predicting COVID-19 severity. This is a common regression method with high-dimensional data (Cox proportion hazard regression model) that has been extended to and broadly used in logistic regression models for outcome forecasting and survival analysis. This approach may be superior to conventional methods when choosing predictors and may allow researchers to combine selected particular features into single signatures [69,70].

Another study demonstrated that machine learning algorithms are superior to traditional statistical modeling approaches for predicting mortality in patients with pneumonia. However, it was found that none of the samples or models assessed showed overall precise predictions of patient mortality, and all the samples revealed wide variations in performance based on the measures used [71].

In a recent study, researchers suggested an algorithm that could anticipate the mortality rate of patients with COVID-19 with accuracy that reached 90%. In that study, machine learning methods were used to establish a predictive model for early recognition of critically sick patients based on clinical and epidemiological data obtained from patients infected with COVID-19. The working mechanism for this machine learning model was based on quantitative sorting of the clinical features according to their criticality. The revealed features were then sorted, and an interpretable clinical route was obtained [23,72].

### AI for COVID-19 Modeling and Simulation

Mathematical modeling of viruses and infections may help simplify the process of understanding virus dynamics. Many authors have used ordinary differential equations in virology and epidemiology to model and simulate different scenarios [73-75].

Viruses are believed to be among the most numerous and divergent biological systems [76]. However, despite their diversity, many shared events and common processes are found in most or possibly all viruses, such as viral replication cycles, which are necessary for productive infection.

Disruption of one or more of these steps may impair or prevent propagation of the virus. Similarly, destabilizing the infective virions before they can attack host cells may be an effective way to prevent propagation. From a therapeutic viewpoint, the process of modeling and simulating the molecular-level dynamics of SARS-CoV-2 in detail at every stage in addition to the virions themselves is important and desirable in such situations and can greatly aid understanding and management of infection with the virus. This knowledge would also be required to address emerging drug-resistant viral strains and future outbreaks of novel pathogenic species similar to COVID-19. In theory, developing therapeutics that can target single or multiple steps in the viral replication cycle or critical processes that have limited capacity for viable mutation can reduce the opportunity for SARS-CoV-2 to develop resistance to administered drugs. Likewise, if simulations aid understanding of the dynamic and structural basis for COVID-19 drug resistance, antiviral drugs can be modified to account for mutations [77].

As in numerous areas in biology, to obtain a comprehensive understanding of virus operations, multidisciplinary approaches are required. Supported by structural biology advancements, AI and computational methods have emerged as very powerful tools that can complement experimental techniques with the use of mathematical modeling and simulations. In several cases, AI and computational approaches can help bridge information gaps among experiments through reporting in different temporal and spatial domains in addition to their considerable predictive powers.

### AI Robotics for Medical Quarantine and Isolated Patients With COVID-19

COVID-19 is a highly transmissible disease that poses a real threat to health care workers. Transmission of this disease to health care workers is highly likely, especially during pandemics when hospitals are overloaded with infected patients.

AI can offer safe and efficient solutions, such as robots that health care professionals can operate while teleconferencing with patients. Teleoperated robots can accomplish common nursing tasks in hazardous areas, such as delivering meals or medications, collecting specimens, and transporting waste, with high accuracy and efficiency [78]. An obvious advantage of using these robots is that a single operator can control multiple robots while rapidly switching between quarantine areas. Other advantages include the ability to communicate with patients via a virtual telepresence system 24 hours per day, 7 days per week. Moreover, a robot called TRINA (Tele-Robotic Intelligent Nursing Assistant) was used to perform error-prone nursing jobs and showed promising results [79].

### Toward Preventative Medicine Using AI and Telemedicine

Several studies have specifically demonstrated the significance of using telemedicine in public health emergencies and disasters. Telemedicine programs take time to develop; however, health systems that have already developed telemedical innovations can leverage and modify them to rapidly manage COVID-19 outbreaks [80].

Forward triage is considered to be a central strategy of health care surge control; it depends mainly on sorting patients prior to their arrival at the emergency department. On-demand or direct-to-consumer telemedicine is a forward triage method that enables effective screening of patients. This screening protects patients, health care workers, and the community from exposure; additionally, it is both patient-centered and conducive to self-quarantine. Telemedicine allows patients and physicians to communicate at any time by using smartphones and webcam-enabled computers [18,81].

At present, the main barrier to large-scale telemedicine screening for COVID-19 is testing coordination. As the availability of testing sites increases, development and integration of local systems into telemedicine workflows is needed to test appropriate patients while decreasing exposure using tents, in-car testing, or dedicated office space. To keep pace with the evolving recommendations regarding COVID-19, health systems are employing bots or automated logic flows that can refer only moderate- or high-risk patients to nurse triage lines and can also allow patients to request video visits with on-demand providers [7]. It is important that practices not routinely refer patients to urgent care medical centers or emergency departments, as this will create exposure risk for health care providers and overload these centers with patients.

Before the outbreak of COVID-19, several emergency departments adjusted their provider-in-triage models for rapid initial testing and evaluation to allow remote providers to perform intake [82]. In emergency situations, web conferencing software with secured open lines from the triage room to a provider can be rapidly implemented [83]. Employing a single remote clinician to cover several sites can address workforce challenges; however, this measure is difficult to implement if the software lacks a queuing function. To avoid exposing staff, telehealth visits can be conducted using paired tablets or commercial systems that enable communication with providers through dedicated connections. However, this system does not fully eliminate exposure of health care providers to patients who require certain manual procedures.

Electronic monitoring programs enable physicians and nurses to remotely monitor patients’ status in several hospitals. Through mobile integrated medical programs or community paramedicine, patients can be managed from their homes, with medical support provided virtually. In Houston, the ETHAN (Emergency Telehealth and Navigation) project uses telemedical oversight to augment in-person care provided by nearly 1000 responders, decreasing the requirement for transportation to emergency departments [84,85].

Telemedicine can offer rapid access to specialists who are not instantly available in person. Barriers to implementing these programs are largely related to credentialing, payment, and specialist staffing [86]. COVID-19 has raised concerns regarding workforce capacity. Telemedicine can enable quarantined physicians to remotely manage and treat patients, freeing time for other physicians to provide in-person care.

In addition, remote training sessions and online training modules can be made available to patients or clinicians who need assistance or in-time training. Program implementation, regulatory and payment structures, credentialing across hospitals, and state licensing will all require time; however, health systems that have already invested in telemedicine are well positioned to ensure that the patients with COVID-19 obtain the care they require. In this instance, telemedicine may be a perfect virtual solution.

## Discussion

AI can potentially provide novel and reliable paradigms for health care services. Due to the nearly unlimited abilities of AI that are gained from its numerous algorithms and approaches, it can help address the virulent spread of the SARS-CoV-2 virus worldwide. Proper application of AI through the use of both existing and novel machine learning approaches may be pivotal to eliminating COVID-19. Furthermore, there is a need for major investment in this field to enable rapid response to the dangers of this disease; this may be a major factor in saving lives worldwide.

## Abbreviations

AI: artificial intelligence
COVID-19: coronavirus disease
CT: computed tomography
EHR: electronic health record
ETHAN: Emergency Telehealth and Navigation
IoIT: Internet of Intelligent Things
IoT: Internet of Things
PDA: personal digital assistant
RT-PCR: reverse transcriptase–polymerase chain reaction
SARS-CoV-2: severe acute respiratory syndrome coronavirus 2
TRINA: Tele-Robotic Intelligent Nursing Assistant
